# Supplementary material for: Resolving the full spectrum of human genome variation using Linked-Reads
Source: Genome Res. 2019 Apr;29(4):635–45. doi: 10.1101/gr.234443.118 (PMC6442396; doi:10.1101/gr.234443.118)
Supplement: Supplemental Material [file supp_gr.234443.118_Supplemental_longranger-2.2.zip › longranger-2.2.tar.bz2!/lr2.2-public/lib/stan/cmdstan/stan/doc/api/html/structstan_1_1gm_1_1corr__matrix__var__decl.html]

Stan: stan::gm::corr\_matrix\_var\_decl Struct Reference


|  |  |
| --- | --- |
|  | Stan  2.5.0  probability, sampling & optimization |


- Main Page
- Related Pages
- Namespaces
- Classes
- Files

- Class List
- Class Index
- Class Hierarchy
- Class Members

All Classes Namespaces Files Functions Variables Typedefs Enumerations Enumerator Friends Macros Pages

- stan
- gm
- corr\_matrix\_var\_decl

Public Member Functions |
Public Attributes |
List of all members

stan::gm::corr\_matrix\_var\_decl Struct Reference

`#include <ast.hpp>`

Inheritance diagram for stan::gm::corr\_matrix\_var\_decl:

|  |  |
| --- | --- |
| Public Member Functions | |
|  | corr\_matrix\_var\_decl () |
|  | |
|  | corr\_matrix\_var\_decl (expression const &K, std::string const &name, std::vector< expression > const &dims) |
|  | |
| Public Member Functions inherited from stan::gm::base\_var\_decl | |
|  | base\_var\_decl () |
|  | |
|  | base\_var\_decl (const base\_expr\_type &base\_type) |
|  | |
|  | base\_var\_decl (const std::string &name, const std::vector< expression > &dims, const base\_expr\_type &base\_type) |
|  | |

|  |  |
| --- | --- |
| Public Attributes | |
| expression | K\_ |
|  | |
| Public Attributes inherited from stan::gm::base\_var\_decl | |
| std::string | name\_ |
|  | |
| std::vector< expression > | dims\_ |
|  | |
| base\_expr\_type | base\_type\_ |
|  | |

## Detailed Description

Definition at line 563 of file ast.hpp.

## Constructor & Destructor Documentation

|  |  |  |  |  |
| --- | --- | --- | --- | --- |
| stan::gm::corr\_matrix\_var\_decl::corr\_matrix\_var\_decl | ( |  | ) |  |

Definition at line 1149 of file ast\_def.cpp.

|  |  |  |  |
| --- | --- | --- | --- |
| stan::gm::corr\_matrix\_var\_decl::corr\_matrix\_var\_decl | ( | expression const & | *K*, |
|  |  | std::string const & | *name*, |
|  |  | std::vector< expression > const & | *dims* |
|  | ) |  |  |

Definition at line 1150 of file ast\_def.cpp.

## Member Data Documentation

expression stan::gm::corr\_matrix\_var\_decl::K\_

Definition at line 564 of file ast.hpp.

---

The documentation for this struct was generated from the following files:

- src/stan/gm/ast.hpp
- src/stan/gm/ast\_def.cpp


---

[ Stan Home Page ]

*© 2011–2014,
Stan Development Team.*
